# Enhancing the Postharvest Stability of Hass Avocado Through Vacuum Impregnation with Antioxidants

**DOI:** 10.3390/foods14213633

**Published:** 2025-10-24

**Authors:** Tania María Guzmán-Armenteros, Armando Echeverría, Jenny Ruales, Maritza Ruiz-Medina, Luis Ramos-Guerrero

**Affiliations:** 1 Carrera de Ingeniería en Alimentos, Facultad de Ingeniería Mecánica y Ciencias de la Producción, Escuela Superior Politécnica del Litoral, Campus Gustavo Galindo, km 30.5 Vía Perimetral, Guayaquil 090902, Ecuador; tamaguzm@espol.edu.ec; 2 Unidad Académica para la Formación Técnica y Tecnológica, Universidad Católica Santiago de Guayaquil, Guayaquil 090615, Ecuador; neptali.echeverria@cu.ucsg.edu.ec; 3 Department of Food Science and Biotechnology, Escuela Politécnica Nacional, Quito 170143, Ecuador; jenny.ruales@epn.edu.ec (J.R.); maritza.ruiz@epn.edu.ec (M.R.-M.); 4 Carrera de Alimentos, Facultad de Industrias Agropecuarias y Ciencias Ambientales, Universidad Politécnica Estatal del Carchi, Tulcán 040102, Ecuador; 5 Grupo de Investigación en Bio-Quimioinformática, Carrera de Ingeniería Agroindustrial, Facultad de Ingeniería y Ciencias Aplicadas, Universidad de Las Américas (UDLA), Quito 170124, Ecuador

**Keywords:** Hass avocado, postharvest, vacuum impregnation, shelf life

## Abstract

This study assessed the effect of vacuum impregnation with antioxidants on the postharvest quality of Hass avocados. Fruits were treated with 1% calcium lactate + 1% ascorbic acid (T1) or 1% calcium lactate + 1% citric acid (T2), under 1 bar for 30 s, and compared with untreated controls. Physicochemical properties, enzymatic activity, color, texture, and sensory quality were monitored during 18 days at 12 °C (±1). Both treatments reduced weight and firmness loss, inhibited polyphenol oxidase activity, and delayed browning. Shelf life was extended to ~9.4 days (T1) and ~10.1 days (T2) compared with 6.99 days in controls. These results show that vacuum impregnation with organic acids moderately improves avocado preservation under refrigerated storage.

## 1. Introduction

The Hass avocado (*Persea americana* Mill.) is one of the most commercially valued tropical fruits worldwide, mainly due to its consumer acceptance and global production volume. In addition, it is recognized for its high content of healthy fatty acids, antioxidants, and bioactive compounds [1,2,3]. However, its rapid ripening and sensitivity to postharvest conditions represent major challenges for its marketing and distribution.

Avocado preservation is compromised by firmness loss, enzymatic browning, and moisture reduction, all of which decrease shelf life and consumer acceptance. Several strategies have been developed to extend postharvest life, including nanocomposite films [4], modified atmosphere packaging [5], biopolymer coatings [6], edible coatings with nanoparticles [7], near-freezing storage [8], evaporative cooling systems [9], and hydrocolloid-based gels [10]. Although effective, these technologies often present limitations such as changes in sensory properties or high implementation costs at the industrial level. Chemical treatments with organic acids and calcium salts have also been reported to reduce browning and maintain texture in avocados [5,6,7], but their application remains restricted by consumer preferences and regulatory aspects.

Vacuum impregnation (VI) is a promising non-thermal technology that enables the incorporation of functional compounds into plant tissues, improving firmness, reducing enzymatic browning, and maintaining structural integrity [11,12]. This technique has been successfully applied in fruits such as apples, mangoes, papayas, and melons [12,13,14], where it has improved texture stability and reduced enzymatic deterioration. During the process, the impregnating liquid diffuses into the tissue voids, promoting uniform distribution of the antioxidant and firming compounds and improving their effectiveness in preserving fruit quality [11,12]. However, its application to avocados has been scarcely explored, despite their unique composition characterized by high lipid content and distinct oxidative metabolism. These particularities suggest that avocado may respond differently to VI compared with other fruits, highlighting the need for specific studies.

Therefore, this study aimed to evaluate the effect of vacuum impregnation with ascorbic and citric acids, combined with calcium lactate, on the preservation of Hass avocados during refrigerated storage. Physicochemical changes, enzymatic activity, color, texture, and estimated shelf life were analyzed to determine the potential of this technology as an alternative for extending the commercial viability of avocados and reducing postharvest losses.

## 2. Materials and Methods

### 2.1. Sample Selection

Hass avocados from local producers of the coast region were selected based on quality parameters to ensure sample consistency and minimize variability in experimental results. These parameters were standardized to ensure uniformity in color, size, weight (250–300 g) and firmness (20–25 N); the latter range was selected because previous studies on Hass avocado have reported that this range corresponds to the stage of physiological maturity where fruits are firm enough to resist handling damage while still acceptable for consumers in terms of texture and flavor [15,16,17]. Only fruits with homogeneous skin and without external defects or visible fungal infection were selected [16]. Ripeness was further verified by measuring dry matter content, which was within 21–24%, the range considered appropriate for Hass avocado at harvest [18].

### 2.2. Treatments

**Figure 1:**
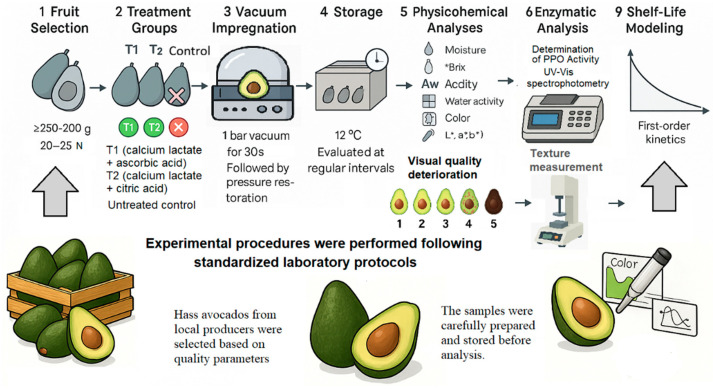
Schematic representation of the experimental design for evaluating the postharvest quality of Hass avocado (*Persea americana* Mill.) subjected to vacuum impregnation treatments. Treatments: Control (untreated), T1 (1% calcium lactate + 1% ascorbic acid), and T2 (1% calcium lactate + 1% citric acid). Fruits were stored at 12 °C (±1) for 18 days.

Two functional solutions were formulated for vacuum impregnation, combining calcium lactate as a firmness-stabilizing agent with organic acids as antioxidants to delay enzymatic browning and preserve the structural integrity of avocado tissues during storage at 12 °C (±1). The control group consisted of untreated fruits (no vacuum impregnation). Treatment 1 (T1) included 1% calcium lactate + 1% ascorbic acid, whereas Treatment 2 (T2) consisted of 1% calcium lactate + 1% citric acid (Figure 1). These formulations were chosen because calcium lactate acts as a firmness-stabilizing agent through interactions with pectic substances, while ascorbic and citric acids function as antioxidants that modulate pH, inhibit polyphenol oxidase.

For each treatment (Control, T1, T2), a total of 35 avocados were used, divided into five biological replicates (n = 5). The storage study lasted 12 days, with measurements performed every two days (7 time points: days 0, 2, 4, 6, 8, 10, 12). At each interval, one fruit per replicate (five fruits per treatment) was randomly selected for analysis, ensuring that all replicates were represented at every time point. This destructive sampling design guaranteed that fruits were not reused once analyzed.

All determinations were carried out in triplicate (technical replicates) to minimize analytical error; however, only biological replicates (n = 5 per treatment and time point) were considered in the statistical analyses (ANOVA and Tukey’s HSD). This design provided robust replication while maintaining consistency across treatments and storage times, helping to ensure reliable results for parameters such as enzyme activity, firmness, and color during refrigerated storage [19,20].

### 2.3. Vacuum Impregnation Process

**Figure 2:**
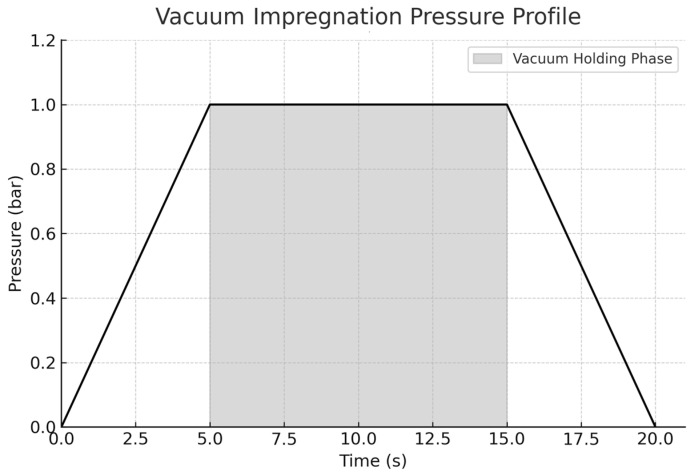
Pressure profile of the vacuum impregnation process used in this study. The curve illustrates a controlled pressure reduction over 5 s, a constant vacuum holding phase at 1 bar for 10 s (shaded area), and a subsequent 5 s restoration to atmospheric pressure. This profile reflects the standard conditions applied during treatment to facilitate liquid uptake and intercellular gas displacement in Hass avocados.

Vacuum impregnation was performed using industrial vacuum sealing equipment at 1 bar for 30 s, following established protocols for the incorporation of stabilizing agents into plant tissues. The vacuum conditions (1 bar for 30 s) were chosen based on prior studies demonstrating that these parameters effectively facilitate liquid penetration in avocado tissues without causing peel cracking (Figure 2) [11,12,21]. The fruits were immersed in the prepared solution and the air was evacuated to induce expansion of the intercellular spaces, facilitating gas elimination and solution absorption [11]. Upon gradual restoration of atmospheric pressure [11,12,13].

### 2.4. Physicochemical Analysis

Physicochemical analyses included moisture content, soluble solids (°Brix), titratable acidity, water activity (Aw), and weight loss, following standardized protocols and AOAC method. Moisture was determined using a thermobalance (Sartorius MA37, Gottingen, Germany; ±0.001 g precision) via the constant weight oven-drying method at 105 °C and expressed as a percentage of mass reduction relative to the initial weight, using Equation (1). Soluble solid content (SSC) was determined using a portable digital refractometer (DiFluid Pocket Refractometer OFT-10, Shenzhen, China), which provided direct °Brix readings of the aqueous phase after separation from the lipid fraction. SSC was calculated according to Equation (2), and titratable acidity was assessed by titration with 0.1 N NaOH and expressed (3) as % citric acid [22]. Water activity (Aw) was measured using a water activity meter (Aqualab Series 3 TE m Decagon Devices Inc., Pullman, WA, USA; ±0.003 Aw accuracy) based on chilled mirror dew point technique, ensuring precision at each evaluation stage. These determinations were monitored by individual measurements at regular intervals (two days) during storage at 12 °C (±1).

(1) $$Wp=\frac{Wo-Wt}{Wo}\times 100$$

where (*Wo*) is the initial weight and (*p*) the weight over time (*t*).

(2) $$SSC=P\;\frac{Mk}{Mk+Ms}$$

where P denotes the refractometer reading (%), while Mk and Ms refer to the masses of the edible portion and the diluted sample, respectively (g).

(3) $$TA=\frac{k+∆V+C+s}{m}$$

where k is the conversion factor (0.064 for citric acid), ∆V is the volume of sodium hydroxide used in the titration (mL), C is the NaOH concentration (mol/L), s is the sample volume, and m is the sample mass (g).

### 2.5. Polyphenol Oxidase (PPO) Activity Analysis

Polyphenol oxidase (PPO) activity was measured via UV-Vis spectrophotometry (Biotek Instruments, Winooski, VT, USA) using 4-methyl catechol (4-MC) as the substrate [19,22]. Avocado pulp (5 g) was homogenized in 20 mL of 0.1 M sodium phosphate buffer (pH 6.5) and centrifuged at 10,000 rpm for 15 min at 4 °C. The resulting supernatant served as the enzyme extract. For the assay, 2.9 mL of 0.05 M of 4-MC (prepared in the same buffer) was combined with 0.1 mL of enzyme extract in a quartz cuvette. Absorbance at 496 nm [19] was recorded every 30 s for 3 min. PPO activity was expressed in units (U/mL), with one unit defined as a change of 0.001 absorbance units per minute. All measurements were performed in triplicate and reported as mean ± standard deviation.

### 2.6. Color and Texture Analysis

Color was assessed using a portable digital colorimeter (Model NH310, Shenzhen 3NH Technology Co., Ltd., Shenzhen, China), operating under the CIE Lab color space. Measurements were taken using standardized D65 illumination and a 10° standard observer, with an 8 mm aperture size. The device recorded lightness (L), red–green (a*), and yellow–blue (b*) values to evaluate color stability and monitor the progression of browning throughout storage [23]. From these primary parameters, three derived indices were calculated: total color difference (ΔE*), chroma (c*), and hue angle (h*). ΔE* was computed using Equation (4) to quantify overall color deviation from the initial state (denoted by subscript zero). Chroma, indicating color saturation, was calculated via Equation (5), while hue angle (h*), describing the perceived tone, was derived from Equation (6) and reported in degrees [21,23].

(4) $$∆E=\sqrt{(L^{*}-{L_{0}}^{*}{)}^{2}+{\left(a^{*}-{a_{0}}^{*}\right)}^{2}+{\left(b^{*}-{\;b_{0}}^{*}\right)}^{2}}$$

(5) $$c^{*}=\sqrt{({a^{*})}^{2}+({b^{*})}^{2}}$$

(6) $$h^{*}=\left(\frac{b^{*}}{a^{*}}\right)$$

Texture was assessed through a uniaxial compression test using a digital texture analyzer (Brookfield CT3, AMETEK Brookfield, Middleboro, MA, USA). For the main firmness evaluation, a 100 mm flat compression plate was employed, as this geometry provides uniform contact with the fruit surface and is considered standard for deformation measurements in whole avocados. Each fruit was placed centrally on the platform, and the plate descended at a constant rate (1 mm/s) until reaching a 4 mm deformation depth. The maximum force (N) required for compression was recorded as an indicator of firmness evolution throughout storage [20,21,23].

### 2.7. Shelf Life Analysis

Shelf life was estimated by applying a first-order kinetic model (2) to the deterioration index (DI) over time, with DI = 45 defined as the critical threshold for loss of acceptability based on texture and sensory evaluation. Using regression analysis in Python (version 3.12.11), the time required for each treatment (T1, T2, and Control) to reach this threshold was calculated. Graphical validation of the model enabled comparison of treatment efficacy in delaying deterioration, and the suitability of the approach was demonstrated by the high coefficients of determination (R^2^ > 0.90) and significant regression fits (*p* < 0.05), confirming the robustness of the shelf life estimation.

(7) $$ln\;DI\;=ln\;{DI}_{c}+k*t$$

where k is the deterioration rate constant obtained from the slope of a linear regression of ln (DI) vs. time.

The deterioration index (DI) was calculated by integrating three key indicators of quality loss: firmness reduction, weight loss, and sensory degradation. Firmness and weight loss were expressed as relative decreases over time, while visual quality deterioration was rated on a standardized 1–5 scale based on external appearance. The scale was defined as follows: 1 = excellent quality (bright green skin, no visible defects); 2 = good quality (slight skin darkening or minimal shriveling); 3 = fair quality (moderate browning and noticeable shriveling, but still acceptable for consumption); 4 = poor quality (severe browning, pronounced shriveling, or initial signs of decay, borderline marketability); 5 = unacceptable (extensive browning, tissue collapse, or evident decay, rejected for sale).

Each parameter was weighted (Wf = 100, Ww = 100, Wd = 10) to reflect its relative impact on overall quality. The Deterioration Index was calculated by integrating these three parameters (firmness, weight loss, and visual quality) using weighting factors (Wf = 100, Ww = 100, Wd = 10). These values were obtained experimentally from preliminary trials with Hass avocados to reflect the visual perception of acceptability and were further supported by previous studies that modeled avocado firmness and quality attributes [15,18]. Both our preliminary results and the literature confirm that firmness and weight loss are the main factors influencing consumer rejection, as they manifest long before visual defects become clearly distinguishable. This composite metric enabled a more holistic evaluation of postharvest degradation and the effectiveness of preservation treatments.

(8) $$DI=\left(\frac{F_{o}-Ft}{Fo}\times W_{f}\right)+\left(\frac{Wo-Wt}{Wo}\times W_{w}\right)+\left(\frac{Dt}{Do}\times W_{d}\right)$$

where F_0_, W_0_, and D_0_ represent the initial values of firmness, weight, and deterioration of the avocados, respectively, while Fₜ, Wₜ, and Dₜ correspond to their values at a given time.

### 2.8. Statistical Analysis

A completely randomized factorial design was implemented to assess the effects of two vacuum impregnation treatments T1 (calcium lactate + 1% ascorbic acid) and T2 (calcium lactate + 1% citric acid) compared to an untreated control, using Hass avocados at maturity stage 4. Samples were randomly assigned and evaluated at two-day intervals during refrigerated storage. All data handling and statistical analysis were performed using Python 3.12.11. The pandas library was employed for data organization and preprocessing, while matplotlib was used to generate high-resolution visualizations. Descriptive statistics were calculated as mean ± standard deviation from three technical replicates per treatment and time point. Biological replicates (n = 5 fruits per treatment) were used as the basis for ANOVA, and pairwise comparisons were performed with Tukey’s HSD test implemented via the statsmodels library. All results are expressed as mean ± standard deviation, and only statistically significant differences (*p* < 0.05) were considered as treatment effects. Graphs were structured in a comparative layout with standardized grayscale tones to distinguish treatments, and included capped error bars to represent variability.

## 3. Results and Discussion

### 3.1. Moisture, Weight Loss, and Water Activities of Hass Avocado

**Figure 3:**
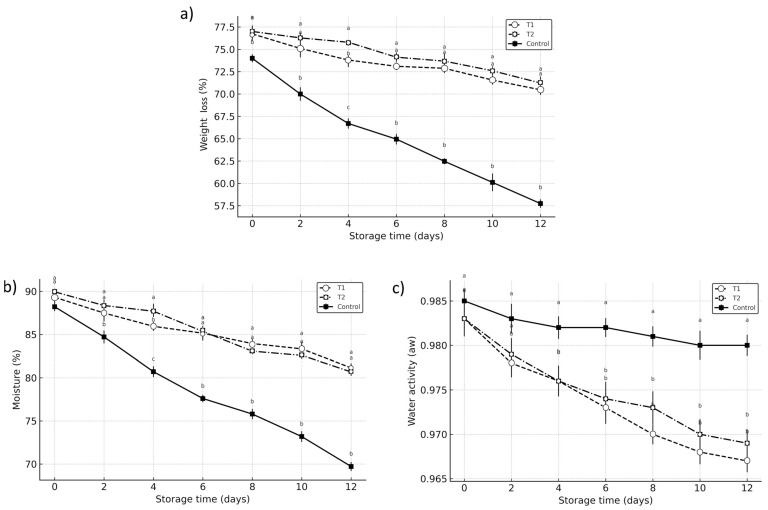
Kinetics of (**a**) weight loss, (**b**) moisture content, and (**c**) water activity (Aw) in Hass avocado during storage at 12 °C (±1). Treatments: Control (untreated), T1 (1% calcium lactate + 1% ascorbic acid), and T2 (1% calcium lactate + 1% citric acid). Values represent means ± SD (n = 5). Different letters indicate significant differences between treatments on each day of storage (*p* < 0.05) according to Compact Letter Display, CLD.

Although all treatments exhibited progressive weight loss during storage, the control group showed the most significant reduction, retaining only 57.74% of its initial weight by day 12, compared to 70.47% in T1 and 71.23% in T2 (Figure 3a). These differences (*p* < 0.05) highlight the role of vacuum impregnation in preserving internal water balance, likely through the integration of osmotic solutes and the formation of semi-permeable barriers that limit transpiration. While T2 maintained slightly higher moisture levels than T1, the difference was not statistically significant (*p* > 0.05). Therefore, the observed advantage of T2 must be interpreted cautiously [24]. These findings align with prior studies indicating that vacuum impregnation can reduce water loss by inducing structural modifications that restrict vapor diffusion and enhance tissue hydration stability.

Moisture content declined in all samples, yet the decrease was significantly more pronounced in the control, dropping from 88.22% to 69.72%, compared to 89.32% to 81.15% in T1 and 89.95% to 80.68% in T2 (Figure 3b). These results suggest that vacuum impregnation treatments contributed to water retention, likely due to improved cellular integrity and reduced vapor permeability (Figure 3b). T1 exhibited slightly better performance than T2 in maintaining higher moisture levels, possibly due to the stabilizing effect of ascorbic acid on membrane permeability [25].

Water activity (Aw) declined significantly (*p* < 0.05) in both T1 and T2 treatments compared to the control during the storage period, although the differences remained subtle. The control consistently exhibited higher Aw values, particularly in the initial days, with a final Aw of 0.980, while T1 and T2 reached 0.965 and 0.974, respectively (Figure 3c). These results suggest that vacuum impregnation (VI) slightly reduced free water availability, potentially contributing to improved microbiological stability and delayed spoilage without compromising overall moisture content [12,13,14].

The reduction in Aw is consistent with prior studies indicating that vacuum impregnation with osmotic solutes such as calcium salts and organic acids can lower water activity by replacing free water with bound solutes and inducing structural modifications in plant tissues [24,25,26]. These solutes help form a semi-permeable matrix that restricts water migration and maintains tissue integrity [27,28]. Moreover, the presence of calcium lactate and citric or ascorbic acid likely contributed to moisture retention and cell wall stabilization, limiting vapor loss and maintaining a more stable water distribution profile [26].

Overall, while Aw remained high across all treatments, a characteristic of high-moisture produces the modest yet statistically significant differences observed, reinforcing the role of vacuum impregnation in subtly modulating water availability. This modulation may reduce microbial susceptibility and support shelf-life extension by preserving structural and biochemical stability in avocado tissues.

### 3.2. Brix/TA, Texture, Degradation and PPO Activity Under Vacuum Impregnation Treatments

**Figure 4:**
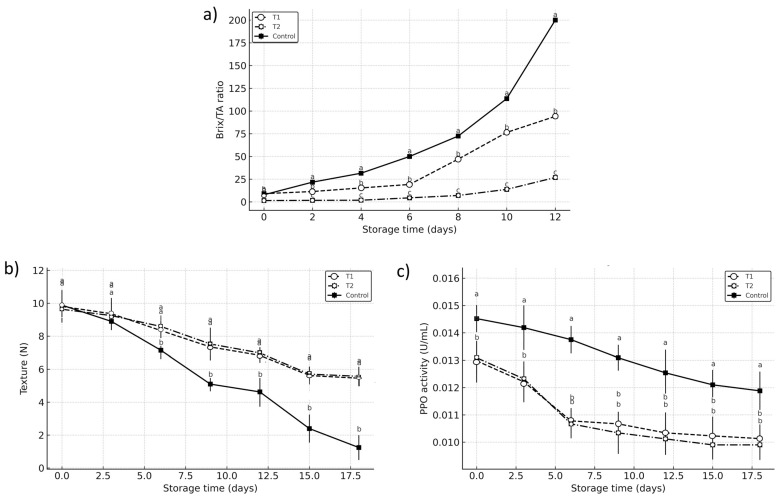
Evolution of (**a**) Brix/TA ratio, (**b**) texture (N), and (**c**) polyphenol oxidase (PPO) activity in Hass avocado during storage at 12 °C (±1). Treatments: Control (untreated), T1 (1% calcium lactate + 1% ascorbic acid), and T2 (1% calcium lactate + 1% citric acid). Values represent means ± SD (n = 5). Different letters indicate significant differences between treatments on each day of storage (*p* < 0.05) according to Compact Letter Display, CLD.

The Brix/TA ratio, a key indicator of ripening, progressively increased across all treatments; however, the control group exhibited a markedly accelerated rise, reaching 200.00 by day 12, compared to 94.12 in T1 and only 26.87 in T2 (Figure 4a). These significant differences (*p* < 0.05) suggest that vacuum impregnation treatments effectively delayed senescence by maintaining titratable acidity and moderating sugar accumulation. T2 demonstrated greater control over the sugar–acid balance, likely due to citric acid’s role in modulating enzymatic activity and respiratory metabolism consistent with prior findings on the use of organic acids to slow ripening in climacteric fruits [25,26,27,28]. Evidence from similar postharvest studies supports this interpretation. Vacuum impregnation has been shown to preserve acidity more effectively than atmospheric methods, as observed in autumn olives and apples [26,27,28,29]. Vacuum conditions restricted solute diffusivity and limited sugar accumulation [26], the isotonic impregnation in apples helped retain natural sugar levels, preventing rapid metabolic shifts [27].

The moderate Brix/TA evolution in treated avocados also reflects the fruit’s slower metabolic response under vacuum impregnation. This contrasts with reports of increased metabolic heat in VI-treated spinach using calcium lactate and sucrose [30,31], underscoring species-specific metabolic differences. Unlike leafy greens, avocados, being climacteric fruits, respond differently to treatment, especially under low-temperature controlled storage (12 °C), which likely contributed to reduced enzymatic activity and respiration, further stabilizing the postharvest condition of the fruit [17].

Regarding firmness (Figure 4b), both T1 and T2 significantly delayed softening compared to the control (*p* < 0.05). On day 12, the control sample lost nearly all structural integrity (1.245 N), while T1 and T2 maintained firmness levels above 5.4 N. Interestingly, T2 consistently preserved slightly higher firmness than T1, albeit not always significantly different (*p* > 0.05). This could imply a stronger calcium-pectin stabilization effect in T2 or a better synergistic behavior of citric acid in preserving the cell wall structure. However, T1’s performance remained comparable, confirming the efficacy of both acids when combined with calcium lactate to reinforce mechanical stability through pectin cross-linking and reduced enzymatic degradation [24,25,26,32]. Some studies report improved crispness in treated fruits and vegetables, an important attribute for products where texture strongly influences consumer acceptance, such as apples and spinach [28,29,30].

Regarding PPO activity, both vacuum impregnation treatments demonstrated a clear inhibitory effect compared to the control (*p* < 0.05), highlighting their role in reducing oxidative enzyme activity and delaying browning processes. By the end of storage, T2 exhibited slightly lower PPO activity (0.0099 U/g) than T1 (0.01013 U/g), although the differences between the two treatments were subtle. This reduction can be attributed to the specific mechanisms of the impregnated compounds: ascorbic acid in T1 acts as a reducing agent, stabilizing phenolic substrates and delaying their oxidation, while citric acid in T2 functions primarily as a metal chelator, binding copper ions essential for PPO catalysis. In addition, vacuum impregnation may have increased the availability of these compounds within avocado tissues, allowing them to interact with endogenous pools of organic acids and reinforcing the fruit’s intrinsic antioxidant system [24,25,26]. This combined action of exogenous supplementation and endogenous reinforcement provides a consistent explanation for the significant inhibition of enzymatic browning observed in treated fruits [19].

### 3.3. Color Properties

**Figure 5:**
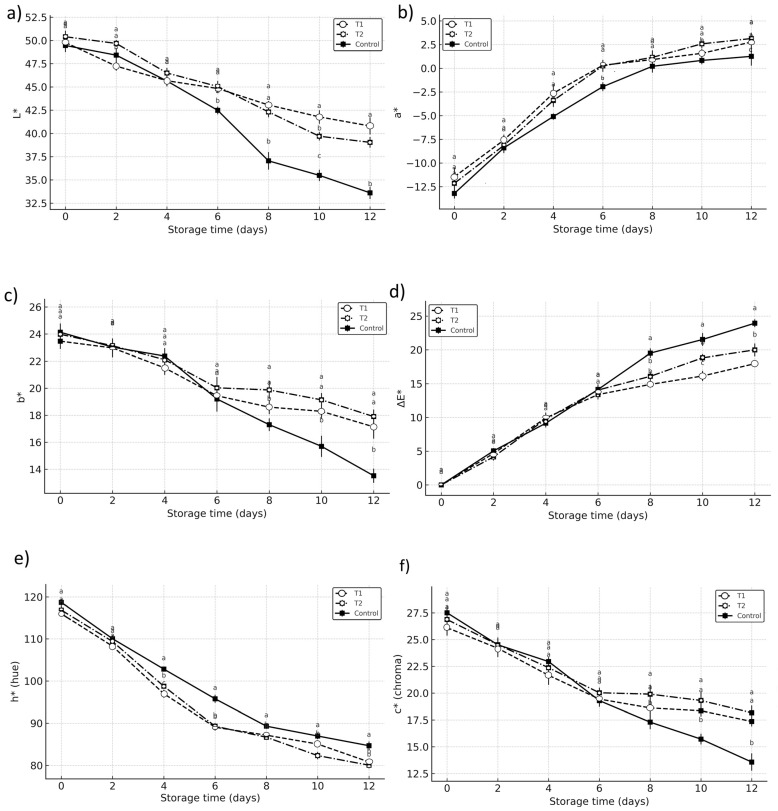
Changes in color parameters (**a**) L* (lightness), (**b**) a* (red–green), and (**c**) b* (yellow–blue), and derived indices (**d**) ΔE*, (**e**) chroma (C*), and (**f**) hue angle (h*) of Hass avocado stored at 12 °C (±1). Treatments: Control (untreated), T1 (1% calcium lactate + 1% ascorbic acid), and T2 (1% calcium lactate + 1% citric acid). Values represent means ± SD (n = 5). Different letters indicate significant differences between treatments on each day of storage (*p* < 0.05) according to Compact Letter Display, CLD.

The progression of color parameters showed significant differences (*p* < 0.05) between vacuum-impregnated fruits and the control during storage. In the control samples, both L* and b* values decreased sharply, from initial readings of 49.46 and 23.8 to 33.61 and 13.7, respectively, indicating pronounced darkening. In contrast, treated fruits preserved higher lightness and yellowness: at the end of storage, T1 showed L* = 40.8 and b* = 17.1, while T2 reached L* = 41.2 and b* = 17.9, confirming that vacuum impregnation reduced the rate of external darkening (Figure 5a,c).

Additionally, the decrease in b* values, indicative of yellowing, were more pronounced in the control (from 24.13 to 13.52) compared to T1 (17.14) and T2 (17.9), reinforcing the idea that vacuum impregnation treatments mitigated enzymatic discoloration. Similarly, significant differences were observed in the chromatic parameter a* (Figure 5b). The increase in a* values, which denotes a shift from green to red tones, was more gradual in T1 and T2 compared to the control. The control sample showed a significant rise from −13.21 to 1.26, whereas T1 and T2 exhibited milder increases (2.77 and 3.14, respectively).

However, analysis of α and hue angle values revealed a different trend. Both T1 and T2 showed a more gradual, but noticeable, shift from green to reddish hues compared to the control. While this could initially be interpreted as a “delayed chlorophyll degradation,” the data could also suggest a more complex explanation. The reddish appearance of treated fruits cannot be entirely attributed to chlorophyll retention; it is more likely related to anthocyanin activation or increased ethylene production induced by vacuum infiltration stress, as previously described in climacteric fruits [12,19,33,34]. Several authors have reported similar responses in other fruits where vacuum impregnation or abiotic stress stimulated pigment biosynthesis and ethylene-related pathways [35,36,37].

Derived indices, such as ΔE and C*, also supported a slower progression of visual impairment in treated samples, although the trend was less consistent across parameters. At day 12, ΔE* values were 17.9 for T1 and 19.9 for T2, compared to 23.9 in the control, while chroma values were better preserved in both treatments. The hue angle decreased in all samples, but the decrease was slightly slower in T1 and T2, reflecting the partial preservation of greenish hues despite the reddish shift [23].

Overall, vacuum impregnation effectively delayed overall darkening, as reflected in L*, b*, ΔE*, and C*, but also induced subtle color changes (a and hue*) that suggest metabolic responses beyond chlorophyll retention. These responses could involve the activation of stress-related pigments or ethylene pathways [12,19,33,34,35,36,37].

It is also important to note that no symptoms of chilling injury, such as skin pitting, flesh browning, or water-soaked areas, were observed in any of the samples during storage at 12 °C (±1). This confirms that the observed differences in color and enzymatic activity were not associated with cold damage but rather with the metabolic responses to vacuum impregnation treatments.

### 3.4. Shelf-Life Determination

Linear regression parameters for the logarithmic deterioration index (ln (DI)) over time for each treatment, deterioration index (ln (DI)) as a function of time (days) for treatments T1, T2, and Control, reported values include the slope, intercept, coefficient of determination (R^2^), *p*-value, and standard error are shown in Table 1. A high R^2^ indicates a strong linear relationship. A deterioration threshold of ln (45) was used to estimate the end of shelf life for each condition.

In the shelf-life analysis, the suitability of the linear regression model was supported by consistently high coefficients of determination (R^2^), indicating that temporal variation explained most of the observed changes in the logarithmic deterioration index (ln (DI)) (Table 1). Furthermore, the low *p*-values obtained across treatments confirm the statistical significance of the fitted models. Together, these metrics confirm the validity and robustness of the linear approach for modeling deterioration dynamics and estimating product shelf life under the conditions evaluated.

**Figure 6:**
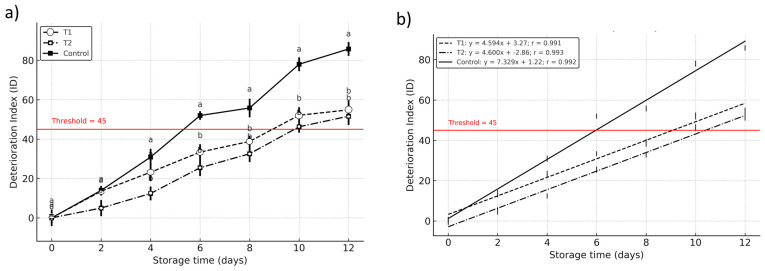
Shelf-life estimation of Hass avocado based on the deterioration index (DI = 45) during refrigerated storage at 12 °C (±1). Treatments: Control (untreated), T1 (1% calcium lactate + 1% ascorbic acid), and T2 (1% calcium lactate + 1% citric acid). Values represent means ± SD (n = 5). Different letters indicate significant differences between treatments on each day of storage (*p* < 0.05) according to Compact Letter Display, CLD. (**a**) Time course of DI for each treatment; the horizontal red line marks the failure threshold (DI = 45). (**b**) Linear regression fits of DI vs. storage time (equations and r shown in the legend); the intersection with DI = 45 provides the estimated shelf-life.

The first-order kinetic modeling of the deterioration index (DI) revealed significant differences in shelf life among treatments, with T1 (calcium lactate + ascorbic acid) and T2 (calcium lactate + citric acid) extending postharvest viability to approximately 9.4 and 10.1 days, respectively, compared to 6.99 days in the untreated control (DI = 45 threshold) (Figure 6). These findings indicate that vacuum impregnation significantly delayed deterioration (*p* < 0.05), particularly in T2, suggesting a stronger protective effect from citric acid’s chelating activity in stabilizing enzymatic and oxidative reactions (Figure 6). While T1 also improved shelf life, the marginally shorter duration may reflect differences in antioxidant mechanism effectiveness under the tested conditions [15,33]. The control group’s early decline highlights the rapid senescence characteristic of untreated Hass avocados under refrigerated (4 °C) storage.

These results are consistent with previous studies reporting the efficacy of vacuum infiltration in improving structural stability and moderating physiological degradation in perishable products. The lower deterioration rates observed in treated samples align with the hypothesis that vacuum impregnation facilitates a more uniform and effective delivery of functional additives into the fruit matrix, potentially reinforcing cell wall integrity and delaying enzymatic browning and tissue softening [12,13,14,36].

The kinetic profile observed in ln (DI) not only reinforces the effectiveness of VI but also provides a quantitative framework for comparing shelf-life performance between treatment strategies. Notably, the combination of VI with bioactive compounds appears to generate synergistic effects, suggesting that beyond physical stabilization, biochemical interactions at the tissue level may contribute to the modulation of stress-sensitive metabolic pathways [35,36,37]. This aligns with the literature advocating for minimally invasive postharvest interventions that extend shelf life without compromising sensory or nutritional quality in commercial handling practices for climacteric fruits [34,35].

A limitation of this study is that the control fruits were not subjected to vacuum impregnation with water alone, which makes it difficult to completely separate the physical effect of vacuum infiltration from the chemical effect of ascorbic and citric acids. However, the fact that both T1 and T2 consistently showed lower PPO activity, delayed softening, and extended shelf life compared with the untreated control suggests that the improvement was not only due to water infiltration but to the specific action of the bioactive compounds. Future studies should include a water-only vacuum control under commercial storage conditions to better discriminate the contribution of the impregnation process itself from that of the antioxidant agents and to evaluate the economic feasibility of this approach in real supply chains.

## 4. Conclusions

Vacuum impregnation with antioxidant solutions significantly reduced weight loss, softening, and enzymatic browning in Hass avocados during refrigerated storage. Treatments extended shelf life from ~7 days in the control to ~9.4 days with calcium lactate + ascorbic acid (T1) and ~10.1 days with calcium lactate + citric acid (T2). Although the extension was only 2–3 days under ideal storage at 12 °C, this improvement is relevant in a real supply chain context, where avocados are typically stored and transported for 7–10 days before reaching retail markets. Even short extensions can reduce postharvest losses, provide greater flexibility in logistics, and improve fruit availability for export and commercialization. These findings confirm that vacuum impregnation with organic acids is a promising non-thermal strategy to improve postharvest quality, though further validation under commercial conditions is needed to optimize its applicability and economic feasibility.

## Figures and Tables

**Table 1:** Linear regression parameters for the logarithmic deterioration index (ln (DI)) over time for each treatment.

Treatment	Slope	Intercept	R_Squared	*p*_Value	Std_Error
T1	0.13686	2.52061	0.92878	0.00195	0.01895
T2	0.22728	1.51151	0.90376	0.00359	0.03708
Control	0.17056	2.61358	0.88454	0.00520	0.03081

## Data Availability

The original contributions presented in this study are included in the article. Further inquiries can be directed to the corresponding author.

## Abbreviations

The following abbreviations are used in this manuscript:

VI	Vacuum impregnation
Aw	Water activity
SSC	Soluble solid content
PPO	Polyphenol oxidase
L	Lightness
a*	Red-green color
b*	Yellow-blue color
ΔE	Color difference
c*	Chroma
h*	Hue angle
